# Three-Dimensional Surface Topography for the Assessment of Spinal Alignment: A Cross-Sectional Study of Biomechanical Correlates

**DOI:** 10.3390/diagnostics16101445

**Published:** 2026-05-09

**Authors:** Brigitte Osser, Csongor Toth, Gyongyi Osser, Laura Ioana Bondar, Liliana-Oana Pobirci, Florin Mihai Marcu, Ramona Nicoleta Suciu, Nicoleta Anamaria Pascalau, Adina Mincic, Corina Dalia Toderescu

**Affiliations:** 1 Doctoral School of Biomedical Sciences, University of Oradea, 410087 Oradea, Romania; osser.brigitte@student.uoradea.ro (B.O.); bondar.lauraioana@student.uoradea.ro (L.I.B.); 2 Faculty of Physical Education and Sport, “Aurel Vlaicu” University of Arad, 310130 Arad, Romania; 3 Department of Biology and Life Sciences, Faculty of Medicine, “Vasile Goldiș” Western University of Arad, 310025 Arad, Romania; 4 Department of Psycho Neuroscience and Recovery, Faculty of Medicine and Pharmacy, University of Oradea, 410087 Oradea, Romania; opobirci@uoradea.ro (L.-O.P.); marcu.florin@didactic.uoradea.ro (F.M.M.); ramona_suciu@uoradea.ro (R.N.S.); nicoleta.pascalau@didactic.uoradea.ro (N.A.P.); 5 Department of Preclinical Sciences, Faculty of Medicine and Pharmacy, University of Oradea, 410087 Oradea, Romania; amincic@uoradea.ro; 6 Department of Pharmaceutical Sciences, Faculty of Pharmacy, “Vasile Goldiș” Western University of Arad, 310045 Arad, Romania; toderescu.corina@uvvg.ro

**Keywords:** adolescents, biomechanical phenomena, postural balance, rehabilitation, spine, surface imaging, three-dimensional

## Abstract

**Background/Objectives**: Spinal alignment is a key determinant of biomechanical function and postural stability, particularly during periods of growth and development. Three-dimensional (3D) surface topography offers a non-invasive method for assessing spinal posture. This study aimed to evaluate spinal alignment parameters in a mixed adolescent and adult population, to investigate sex-related differences, and to analyze biomechanical relationships between spinal components. **Methods**: A total of 98 participants (aged 11–45 years) underwent 3D spinal surface topography assessment. Descriptive statistics were calculated for sagittal, coronal, and rotational parameters. Group comparisons between sexes were performed using independent samples *t*-tests. Pearson correlation analysis and linear regression were used to assess the relationships between spinal parameters. Logistic regression analysis was conducted to identify predictors of clinically relevant rotational asymmetry (surface rotation RMS > 6°). **Results**: Most participants exhibited near-physiological sagittal alignment, with thoracic kyphosis and lumbar lordosis within normal ranges. However, approximately 20% demonstrated clinically relevant rotational asymmetry. Female participants showed significantly higher rotational asymmetry compared to males (*p* = 0.008), while sagittal parameters did not differ significantly. Strong correlations were observed between thoracic kyphosis and cervical sagittal displacement (r = 0.77). Rotational asymmetry was negatively correlated with sagittal parameters and significantly predicted coronal imbalance (β = 0.38, *p* < 0.01; R^2^ = 0.21). **Conclusions**: 3D surface topography provides a non-invasive method for assessing external postural alignment and surface-based asymmetries. Rotational asymmetry appears to represent a relevant component of spinal imbalance and is associated with coronal deviation within a multi-planar framework. These findings support the use of integrated biomechanical assessment in the evaluation of spinal alignment.

## 1. Introduction

Spinal alignment plays a fundamental role in maintaining biomechanical efficiency, postural stability, and overall musculoskeletal health. Alterations in spinal curvature and posture have been associated with pain, functional limitations, and reduced quality of life across different age groups, particularly during periods of rapid growth such as adolescence [1,2,3,4]. Early identification of postural deviations is therefore essential for prevention and timely intervention.

Traditional methods for evaluating spinal alignment, including clinical examination and radiographic imaging, present certain limitations. Clinical assessment may lack sensitivity for detecting subtle or early-stage deviations, while radiographic techniques, although considered a reference standard, involve exposure to ionizing radiation and are not suitable for repeated screening, especially in young populations. In this context, three-dimensional (3D) surface topography has emerged as a non-invasive and radiation-free alternative, enabling detailed assessment of spinal alignment in multiple planes [5,6,7,8,9,10].

Among these approaches, rasterstereographic systems—such as the DIERS Formetric III 4D—have been widely used for the non-invasive evaluation of spinal posture. These systems reconstruct the dorsal surface of the trunk and estimate spinal alignment parameters with good reliability and reproducibility. Previous studies have demonstrated a strong correlation between surface topography measurements and radiographic parameters, supporting their clinical applicability in both screening and follow-up contexts [9,11,12,13].

Previous studies have demonstrated that mild postural deviations are common in adolescent and young adult populations, reflecting ongoing musculoskeletal development and adaptation [4,14]. Furthermore, spinal alignment is increasingly recognized as a 3D phenomenon, involving complex interactions between sagittal, coronal, and rotational components. The concept of biomechanical interdependence suggests that changes in one spinal segment or plane may influence alignment in others, emphasizing the need for integrated analysis [15,16,17].

Despite these advances, several gaps remain in the current literature. First, many studies focus on isolated spinal parameters rather than integrated, multi-planar assessment. Second, the relationship between rotational asymmetry and other alignment parameters, particularly its predictive role in postural imbalance, remains insufficiently explored. Third, existing studies are often limited to specific age groups, particularly adolescents, reducing the generalizability of findings across broader populations.

Although the use of 3D surface topography in spinal assessment has increased, most existing studies have focused on isolated anatomical parameters or restricted cohorts. The role of rotational asymmetry within a multi-planar framework, particularly in heterogeneous populations that include both adolescents and adults, remains insufficiently characterized.

Therefore, the present study aims to provide a comprehensive evaluation of spinal alignment using 3D surface topography in a heterogeneous cohort of adolescents and adults. Specifically, the study investigates sex-related differences, examines biomechanical relationships between spinal parameters, and evaluates the predictive role of rotational asymmetry within a multi-planar model.

## 2. Materials and Methods

### 2.1. Study Design and Setting

This study was designed as an observational, cross-sectional analysis of spinal alignment parameters obtained using 3D surface topography. Data were collected between December 2024 and February 2026.

Assessments were performed within the facilities of Aurel Vlaicu University of Arad, where spinal measurements were conducted using a non-invasive 3D surface topography system. Follow-up evaluation and rehabilitation programs were carried out in collaboration with Davima Clinic, Arad, and Izvorul Sănătății Medical Center, Arad.

### 2.2. Participants

The study included 98 participants aged between 11 and 45 years. The cohort comprised both adolescents and adults, allowing for the evaluation of spinal alignment across different developmental stages. This approach was chosen to reflect real-world clinical screening populations, where individuals of varying ages present for postural assessment. Given the heterogeneous age distribution, age was incorporated as a covariate in regression analyses to partially account for developmental variability.

Participants were recruited through clinical screening and voluntary participation from individuals referred for postural evaluation. All participants underwent 3D surface topography assessment as part of routine postural screening or clinical evaluation.

Participants were eligible for inclusion if they met the following criteria:

- Age between 11 and 45 years;
- Ability to maintain an upright standing position during assessment;
- Completion of the full 3D spinal surface topography evaluation, including all measured parameters (sagittal, coronal, and rotational);
- Provision of written informed consent. For participants under 18 years of age, written informed consent was obtained from a parent or legal guardian, along with participant assent.

Exclusion criteria included the following:

- Known severe spinal pathology requiring surgical intervention;
- Acute musculoskeletal pain (cervical, thoracic, or lumbar) that could interfere with posture or measurement accuracy;
- Diagnosed neurological disorders affecting postural control;
- Inability to complete the full assessment protocol or maintain the required position during measurement;
- Incomplete datasets, defined as missing key spinal parameters required for analysis.

**Figure 1:**
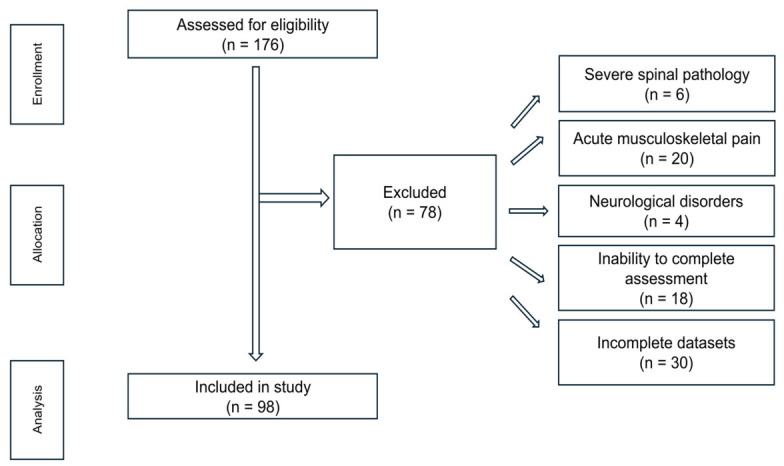
Flowchart of participant selection, eligibility, and analysis process.

The participant selection process and flow, based on the applied inclusion and exclusion criteria, are presented in Figure 1.

The inclusion of both adolescents and adults was intentional in order to capture a broader spectrum of spinal alignment patterns across different stages of musculoskeletal development. This approach allows for the exploration of generalizable biomechanical relationships that may persist across age groups, although age-related variability is acknowledged.

### 2.3. Spinal Assessment and Data Acquisition

Spinal alignment was assessed using a 3D surface topography system (Spine 3D, Sensor Medica, Guidonia Montecelio, Italy), which employs rasterstereography to reconstruct the dorsal surface and estimate spinal parameters without exposure to ionizing radiation.

Measurements were performed with participants in a standardized upright position. The system automatically identified anatomical landmarks, including the vertebra prominens (VP) and the dimples of Venus (DM), enabling the calculation of spinal length and alignment parameters. Data acquisition and analysis were performed using the manufacturer’s dedicated software (version 1.2.4.31). Surface rotation RMS represents the rotation of the dorsal trunk surface and reflects external asymmetry of the thoracic region. It does not provide a direct measure of vertebral rotation and may be influenced by rib cage morphology, thoracic shape, and soft tissue distribution.

The following parameters were analyzed: sagittal spinal inclination (°), cervical sagittal arrow (mm), lumbar sagittal arrow (mm), thoracic kyphosis (°), lumbar lordosis (°), coronal imbalance (mm), trunk imbalance (mm), shoulder obliquity (mm), pelvic obliquity (mm), and surface rotation root mean square (RMS) (°).

Rotational asymmetry was further categorized using a clinically relevant threshold of >6°.

The study focused exclusively on non-invasive surface measurements, and no radiographic imaging was performed as part of the study protocol.

### 2.4. Clinical Management Context

Participants identified with postural deviations during routine clinical evaluation were referred for individualized rehabilitation management, including physiotherapy and postural correction exercises, conducted at Davima Clinic and Izvorul Sănătății Medical Center.

These procedures were part of standard clinical practice and were not included in the study design or analyzed within the scope of the present cross-sectional investigation. No longitudinal follow-up data or intervention outcomes were evaluated.

### 2.5. Statistical Analysis

Statistical analysis was performed using standard descriptive and inferential methods. All analyses were conducted using IBM SPSS Statistics (Version 26.0; IBM Corp., Armonk, NY, USA).

No formal a priori sample size calculation was performed, as this study was designed as an exploratory cross-sectional analysis based on the consecutive inclusion of eligible participants during the study period. This pragmatic approach reflects real-world clinical screening conditions. The sample size was considered sufficient for detecting moderate effect sizes in correlation and regression analyses.

Descriptive statistics included mean values, standard deviations, and ranges for all measured parameters. Group comparisons between male and female participants were performed using independent samples *t*-tests.

Pearson correlation coefficients were calculated to assess the relationships between spinal parameters. Linear regression analysis was conducted to evaluate the predictive value of rotational asymmetry (surface rotation RMS) for coronal imbalance.

Additionally, logistic regression analysis was performed to identify predictors of clinically relevant rotational asymmetry (defined as RMS > 6°). This threshold was selected based on previously reported reference values in surface topography studies, where similar cutoffs have been used in postural screening to distinguish between physiological variation and potentially clinically relevant asymmetry.

To account for potential age-related effects, age was included as a covariate in both linear and logistic regression models. However, other potential confounding variables, such as body composition and sex-specific differences, were not included in the multivariate models.

A *p*-value < 0.05 was considered statistically significant.

### 2.6. Ethical Considerations

The study was conducted in accordance with the principles of the Declaration of Helsinki. Ethical approval was obtained from the Research Ethics Committee of Aurel Vlaicu University of Arad (Approval No. 564/7 September 2024). Additional approvals were obtained from the participating clinical centers, including Davima Clinic (Approval No. 172/1 November 2024) and Izvorul Sănătății Medical Center (Approval No. 138/18 November 2024).

All participants provided written informed consent prior to inclusion in the study. For participants under 18 years of age, written informed consent was obtained from a parent or legal guardian, along with participant assent. All data were anonymized prior to analysis.

## 3. Results

### 3.1. Study Population Characteristics

The study included 98 participants who underwent 3D surface topography assessment of the spine. The cohort was heterogeneous, comprising both adolescents and adults, thereby allowing for the evaluation of spinal alignment across different developmental stages. Most participants were in the adolescent and young adult age range, which is particularly relevant for postural screening and longitudinal follow-up during periods of active growth.

The age of the participants ranged from 11 to 45 years, with a mean age of 19.87 ± 7.23 years. Anthropometric analysis showed a mean height of 166.55 ± 11.12 cm and a mean body weight of 68.73 ± 18.11 kg. These findings indicate moderate variability within the study population, likely reflecting the inclusion of participants at different stages of somatic development.

The mean spinal length measured along the VP–DM axis was 439.87 ± 46.42 mm, with values ranging from 346 mm to 498 mm. Overall, the descriptive data support the characterization of the study sample as a mixed developmental cohort suitable for the evaluation of postural and spinal alignment parameters (Table 1).

Given the heterogeneous age distribution, the reported values represent aggregated patterns and should be interpreted as reflecting overall trends rather than age-specific normative data.

### 3.2. Global Spinal Alignment Parameters

The analysis of spinal parameters obtained using the 3D surface topography system revealed that the majority of participants exhibited near-physiological sagittal alignment.

The mean sagittal inclination of the spine was 1.25° ± 3.49°, indicating an overall neutral posture at the group level. However, individual values ranged from marked anterior to posterior inclinations, reflecting variability in postural control among participants.

The cervical sagittal arrow (45.03 ± 17.47 mm) and lumbar sagittal arrow (42.21 ± 13.18 mm) demonstrated moderate anterior displacement. These findings may reflect adaptive postural patterns observed within the studied population.

Physiological spinal curvatures were generally preserved. The mean thoracic kyphosis angle was 42.12 ± 8.93°, while the mean lumbar lordosis angle was 42.77 ± 12.06°, both falling within ranges typically considered normal.

In the coronal plane, mild deviations were observed. The mean coronal imbalance was −6.03 ± 7.98 mm, indicating slight lateral displacement in some individuals. In contrast, trunk imbalance remained minimal (0.77 ± 1.37 mm), suggesting that global trunk alignment relative to the vertical axis was largely maintained.

Rotational asymmetry, assessed using surface rotation RMS values, averaged 4.02 ± 4.21°. While most participants exhibited low levels of rotational deviation, a subset showed markedly elevated values exceeding 20°, which may reflect postural asymmetries or potential early alterations in trunk alignment, although structural deformity cannot be determined using surface measurements alone.

A detailed descriptive summary of the spinal alignment parameters is presented in Table 2.

**Figure 2:**
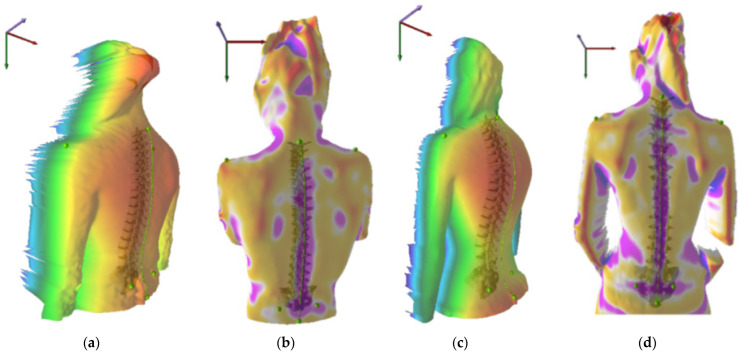
Representative examples of spinal surface topography findings: (**a**) near-physiological sagittal and coronal alignment; (**b**) mild postural deviation with minor asymmetry; (**c**) pronounced rotational asymmetry of the trunk (surface rotation RMS ≈ 21°); (**d**) marked coronal imbalance (≈17 mm), associated with altered sagittal profile, including increased lumbar lordosis (≈76°). Color gradients represent variations in trunk surface contour and asymmetry generated by the rasterstereographic system. The coordinate arrows indicate spatial orientation and anatomical reference planes.

Representative examples of 3D spinal surface topography are illustrated in Figure 2, demonstrating variations from near-physiological alignment to clinically significant postural deviations. The images highlight differences in sagittal contour, coronal alignment, and rotational asymmetry across representative cases. Severe cases included pronounced rotational asymmetry (surface rotation RMS ≈ 21°), increased lumbar lordosis (≈76°), and marked coronal imbalance (≈17 mm).

### 3.3. Sex-Related Differences in Spinal Alignment

To evaluate potential sex-related differences in spinal alignment, comparative analyses were performed between male and female participants.

No statistically significant differences were identified in thoracic kyphosis or lumbar lordosis angles between sexes, indicating that sagittal spinal curvature was comparable across male and female participants within the studied cohort.

In contrast, a statistically significant difference was observed in rotational asymmetry. Female participants demonstrated higher surface rotation RMS values compared to males, suggesting increased rotational trunk asymmetry. This finding may reflect sex-related differences in postural control mechanisms, ligamentous laxity, or neuromuscular adaptation patterns.

The comparative results for the main spinal parameters are summarized in Table 3.

### 3.4. Correlation Analysis Between Spinal Parameters

Pearson correlation analysis was performed to evaluate the relationships between key spinal alignment parameters.

A strong positive correlation was identified between thoracic kyphosis and cervical sagittal displacement (r = 0.77), indicating that increased thoracic curvature is associated with anterior translation of the cervical spine. This finding may be consistent with the concept of functional interdependence between spinal segments; however, it should be interpreted as a statistical association rather than evidence of a causal or functional relationship.

Moderate positive correlations were observed between thoracic kyphosis and lumbar lordosis (r = 0.53), suggesting that physiological spinal curvatures may operate as part of an integrated biomechanical chain. Additionally, lumbar lordosis demonstrated moderate correlations with both cervical sagittal displacement and lumbar sagittal arrow values, which may reflect patterns of alignment across spinal segments.

In contrast, rotational asymmetry (surface rotation RMS) showed negative correlations with several sagittal parameters, including thoracic kyphosis (r = −0.48) and cervical sagittal displacement (r = −0.52). These findings suggest that rotational trunk deviations may develop independently of sagittal curvature alterations and could represent distinct postural patterns.

Overall, the observed correlations should be interpreted as statistical associations and do not establish causal relationships or underlying biomechanical mechanisms.

The full correlation matrix is presented in Table 4.

### 3.5. Regression Analysis of Spinal Parameters

To further explore the relationship between spinal parameters, an age-adjusted linear regression analysis was performed to assess whether rotational asymmetry, expressed as surface rotation RMS, predicts coronal imbalance.

The results indicated that surface rotation RMS remained a significant predictor of coronal imbalance after adjustment for age (β = 0.38, *p* < 0.01), suggesting that increased rotational deviation is associated with greater lateral displacement of the trunk, independent of age-related effects.

Age was included in the model as a covariate and did not emerge as a statistically significant predictor of coronal imbalance (β = 0.12, *p* = 0.09).

The model demonstrated a moderate explanatory capacity (R^2^ = 0.21), corresponding to a medium-to-large effect size (Cohen’s f^2^ = 0.27), as estimated using G*Power 3.1. This indicates that approximately 21% of the variance in coronal imbalance could be explained by the variables included in the model.

These findings suggest that the relationship between rotational asymmetry and coronal imbalance is robust and not primarily explained by age-related variability within the study population.

The results of the regression analysis are summarized in Table 5.

### 3.6. Distribution of Postural Deviations

The distribution analysis of spinal alignment parameters indicated that most participants presented mild postural deviations, particularly within the sagittal plane. Sagittal inclination values were predominantly clustered around neutral alignment, suggesting that the majority of individuals maintained overall postural balance despite minor variations.

Approximately one-fifth of the participants exhibited rotational asymmetry exceeding 6° (surface rotation RMS), which may be considered clinically relevant and could represent early indicators of postural imbalance or incipient structural deviation. These findings highlight the potential utility of 3D surface topography in identifying subclinical alterations that may not yet be evident through standard clinical examination.

In addition to rotational deviations, a subset of participants demonstrated increased values in parameters such as shoulder and pelvic obliquity, suggesting asymmetrical loading patterns in the coronal plane.

Outliers were identified in certain variables, including extreme pelvic inclination values. These were considered likely attributable to measurement artifacts or data acquisition variability and were excluded from further statistical normalization to improve the robustness of the analysis.

Severity categories were defined based on clinically relevant thresholds specific to each parameter. The distribution of postural deviations according to these categories is presented in Table 6.

### 3.7. Logistic Regression Analysis of Rotational Asymmetry

An age-adjusted logistic regression analysis was performed to explore potential predictors of clinically relevant rotational asymmetry, defined as surface rotation RMS > 6°.

Postural parameters, including coronal imbalance and sagittal alignment measures, were considered as potential predictors, with age included as a covariate to account for potential age-related effects.

The analysis suggested that increased coronal imbalance (OR = 1.11, *p* = 0.02) and sagittal deviations (OR = 1.07, *p* = 0.03) were associated with a higher likelihood of rotational asymmetry exceeding the clinical threshold. Lumbar lordosis showed a trend toward significance (OR = 1.04, *p* = 0.08).

Age did not emerge as a statistically significant predictor of rotational asymmetry (OR = 1.02, *p* = 0.21).

These findings indicate a potential association between combined alterations in multiple planes of spinal alignment and clinically relevant rotational deviations, independently of age-related variability.

The results of the logistic regression analysis are summarized in Table 7.

## 4. Discussion

The present study provides a comprehensive evaluation of spinal alignment using 3D surface topography, integrating biomechanical and statistical analyses. The findings support the initial hypotheses and further emphasize the multi-planar nature of postural assessment.

### 4.1. Global Spinal Alignment and Population Characteristics

The present findings indicate that most participants exhibited near-physiological sagittal spinal alignment, with thoracic kyphosis and lumbar lordosis values remaining within ranges generally reported in the literature [18,19,20]. This observation is consistent with the study objectives and suggests that the analyzed cohort did not present major structural sagittal deformities, but rather mild postural variations. This pattern may reflect adaptive postural strategies during growth and early adulthood, where neuromuscular control mechanisms compensate for minor deviations in order to maintain global balance.

Such findings are consistent with previous studies showing that minor deviations are common in adolescent and young adult populations, particularly during periods of active growth and musculoskeletal adaptation [14,21]. In this context, these variations may reflect physiological adaptation rather than fixed pathology, although they may still require monitoring over time.

These findings are also consistent with previous studies using rasterstereographic systems, particularly the DIERS Formetric III 4D, which have demonstrated reliable and reproducible measurements of spinal alignment parameters in both clinical and screening settings. These systems have demonstrated good agreement with radiographic methods for the assessment of sagittal and coronal alignment, supporting their use as non-invasive tools for postural evaluation and longitudinal monitoring [12,22,23].

The variability observed in sagittal inclination and spinal length reflects the heterogeneous composition of the study population, including participants at different developmental stages. These results highlight the importance of individualized assessment in postural screening, as group averages may mask clinically relevant deviations at the individual level [24,25].

Importantly, the present study contributes to the literature by providing an integrated multi-planar biomechanical assessment of spinal alignment in a heterogeneous cohort. The inclusion of a mixed developmental population may also enhance the ecological validity of the findings, reflecting real-world screening conditions.

### 4.2. Rotational Asymmetry and Clinical Relevance

Although mean rotational values were relatively low, a subset of participants demonstrated clinically relevant rotational asymmetry exceeding 6°, with some cases surpassing 20°. These findings are consistent with established screening thresholds for early spinal deformities, including scoliosis [26,27,28,29]. This finding may reflect early-stage alterations in trunk muscle symmetry or neuromuscular control, which have been proposed as contributing factors in the development of rotational spinal deviations.

The identification of approximately one-fifth of the participants with elevated rotational asymmetry highlights the potential of 3D surface topography to identify surface-based asymmetries that may not be evident during routine clinical examination; however, these findings should be interpreted as external postural characteristics rather than direct indicators of structural deformity [9,30,31]. This supports the role of non-invasive imaging techniques in early detection and longitudinal monitoring of postural abnormalities.

### 4.3. Biomechanical Interdependence of Spinal Parameters

The correlation analysis revealed strong and moderate associations between sagittal spinal parameters, particularly between thoracic kyphosis and cervical sagittal displacement, as well as between thoracic kyphosis and lumbar lordosis. These findings may be consistent with the concept of the spine as a functional kinetic chain; however, they should be interpreted with caution, as correlation analysis does not establish causality or underlying biomechanical mechanisms [32,33,34,35,36]. One possible explanation for these associations may involve compensatory adjustments within the spinal kinetic chain, where alterations in one segment lead to adaptive changes in adjacent regions to preserve overall postural equilibrium and minimize mechanical stress.

In contrast, rotational asymmetry demonstrated negative correlations with sagittal parameters, suggesting that axial deviations may follow partially independent patterns relative to sagittal alignment. This observation is in line with the study hypothesis and aligns with previous research emphasizing the 3D nature of spinal deformities [37,38,39,40]. From a clinical perspective, these findings highlight the importance of evaluating spinal alignment across multiple planes rather than relying on isolated parameters.

A key contribution of the present study lies in identifying rotational asymmetry as a clinically relevant parameter within a multi-planar framework. While previous research has primarily focused on sagittal or coronal alignment independently, our findings suggest that rotational components may be associated with postural imbalance and could be considered in comprehensive screening strategies.

Importantly, the observed associations between rotational asymmetry and coronal imbalance remained significant after adjustment for age in both linear and logistic regression models. This suggests that the identified relationships are not solely driven by age-related variability within the study population. However, these findings should also be interpreted in the context of potential residual confounding, as not all relevant variables—such as body composition and sex-specific differences—were included in the multivariate models.

Although the regression model demonstrated a moderate explanatory capacity, a substantial proportion of the variance in coronal imbalance remains unexplained. This suggests that additional factors not included in the present analysis—such as anthropometric characteristics or other biomechanical variables—may contribute to postural variability.

These findings should be interpreted as statistical associations and do not establish underlying biomechanical interdependence or causal mechanisms. They may indicate patterns consistent with a multi-planar and functionally integrated model of spinal alignment.

Logistic regression analysis further identified factors associated with clinically relevant rotational asymmetry (RMS > 6°). It should be noted that the selected threshold (RMS > 6°) represents an operational definition based on prior literature and screening practice, and its diagnostic sensitivity and specificity were not directly evaluated in the present study. These findings suggest that combined alterations across multiple planes may be associated with an increased likelihood of rotational asymmetry; however, they should be interpreted as statistical associations rather than evidence of causal relationships.

### 4.4. Clinical Interpretation of Sex-Related Differences

The present study identified no significant sex-related differences in sagittal spinal curvatures, which is consistent with previous findings indicating similar kyphotic and lordotic profiles between males and females in general populations [20,41,42,43].

However, female participants exhibited significantly higher levels of rotational asymmetry. This observation is in line with epidemiological data demonstrating a higher prevalence of spinal deformities, including scoliosis, among females. Potential contributing factors include differences in ligamentous laxity, neuromuscular control, and growth patterns [44,45]. These findings highlight the importance of considering sex-specific factors in postural assessment and screening strategies.

### 4.5. Predictive Value of Rotational Asymmetry

The regression analyses further supported the biomechanical relevance of rotational parameters. Surface rotation RMS was identified as a significant predictor of coronal imbalance, supporting the proposed analytical model. This finding suggests that rotational deviations may contribute to lateral displacement of the trunk and overall postural instability.

Additionally, the logistic regression model indicated that combined alterations in sagittal and coronal parameters are associated with an increased likelihood of clinically relevant rotational asymmetry. These results reinforce the concept that spinal deformities should be assessed as integrated, multiplanar phenomena rather than isolated parameters [38,46,47]. From a clinical perspective, these findings support the use of rotational parameters as complementary indicators in the assessment and monitoring of postural deviations.

### 4.6. Clinical Implications

The findings of the present study have several important clinical implications. First, 3D surface topography appears to be a valuable, non-invasive tool for the early detection and monitoring of postural deviations, particularly in adolescent populations undergoing rapid musculoskeletal development. Its ability to identify subtle, subclinical alterations supports its use in screening programs and longitudinal follow-up.

Second, the identification of multi-planar relationships between spinal parameters reinforces the need for a comprehensive approach to postural assessment. Rather than focusing on isolated parameters, clinicians should consider sagittal, coronal, and rotational components as part of a unified biomechanical system, which may improve diagnostic accuracy and inform the future development of individualized rehabilitation strategies.

These findings support the use of integrated biomechanical assessment in clinical practice and highlight the potential value of incorporating multiplanar analysis into routine postural evaluation.

### 4.7. Limitations

Several limitations of the present study should be acknowledged. First, no formal a priori sample size calculation was performed. Although the study included 98 participants, the sample size may have limited the statistical power to detect smaller effects, particularly in subgroup analyses. However, based on the observed regression model (R^2^ = 0.21), corresponding to a medium-to-large effect size (Cohen’s f^2^ = 0.27), the study was likely adequately powered to detect moderate associations. Therefore, non-significant findings should be interpreted with caution, as they may reflect limited statistical power rather than the true absence of an effect. In addition, the moderate explanatory power of the regression models (R^2^ = 0.21) indicates that a substantial proportion of the variance remains unexplained, suggesting that additional relevant factors were not captured in the present analysis. Additionally, the threshold used to define clinically relevant rotational asymmetry (RMS > 6°) was based on literature-derived reference values and was not specifically validated in the present study, which may limit its diagnostic precision.

Second, the study population was heterogeneous, including participants across a wide age range (11–45 years) and different stages of musculoskeletal development. Spinal alignment parameters are known to vary with age due to growth-related changes, neuromuscular maturation, and degenerative processes. Although age was included as a covariate in the regression models to account for potential confounding effects, residual age-related variability cannot be fully excluded. In addition, other potential confounding variables, such as body composition and sex-specific differences, were not included in the regression models and may have influenced the observed associations.

Third, the cross-sectional design of the study limits the ability to assess temporal relationships or the progression of postural deviations over time.

An additional limitation relates to the interpretation of surface rotation RMS. This parameter reflects external trunk surface asymmetry rather than direct vertebral rotation and may be influenced by rib cage morphology, thoracic shape, and soft tissue distribution. Therefore, caution is required when interpreting surface rotation as an indicator of underlying structural spinal rotation.

Finally, although 3D surface topography provides valuable non-invasive information on external trunk morphology, it does not replace radiographic assessment and does not directly assess vertebral alignment or quantify parameters such as the Cobb angle. The absence of validation against radiographic reference standards represents an additional limitation of the study. Therefore, the findings should be interpreted as reflecting surface-based postural characteristics rather than structural spinal deformities. Furthermore, factors such as body composition, soft tissue characteristics, and individual anatomical variability may influence measurement accuracy and represent potential sources of variability that were not specifically analyzed in the present study.

### 4.8. Future Directions

Future research should focus on longitudinal study designs to evaluate the evolution of postural deviations during growth and to assess potential changes over time.

The inclusion of radiographic validation in selected cases would allow for direct comparison between surface topography and structural spinal parameters, thereby improving the interpretability and clinical relevance of the findings.

Moreover, incorporating relevant anthropometric variables, such as body composition, would help clarify their role as potential confounders in postural assessment.

Future studies should also incorporate age-stratified analyses and larger, more homogeneous cohorts to better account for developmental differences in spinal alignment.

Finally, combining surface topography with complementary diagnostic modalities may further enhance the accuracy of postural assessment and contribute to more individualized and preventive approaches in clinical practice.

## 5. Conclusions

The present study provides a comprehensive evaluation of spinal alignment using 3D surface topography in a mixed adolescent–adult population. Most participants exhibited near-normal sagittal alignment; however, a notable proportion presented clinically significant rotational asymmetry, highlighting the importance of early screening and monitoring.

The findings confirm the biomechanical interdependence of spinal parameters and emphasize the role of rotational asymmetry as a key component in postural evaluation within a multiplanar framework.

Overall, these results support a comprehensive biomechanical approach to postural assessment, which may contribute to improved early detection and more individualized evaluation strategies in clinical practice.

## Figures and Tables

**Table 1:** Demographic and anthropometric characteristics of the study population (*n* = 98).

Parameter	Mean	SD	Minimum	Maximum
Age (years)	19.87	7.23	11	45
Height (cm)	166.55	11.12	145	192
Weight (kg)	68.73	18.11	32	119
Spine length VP–DM (mm)	439.87	46.42	346	498

**Table 2:** Descriptive statistics of spinal posture parameters.

Parameter	Mean	SD	Minimum	Maximum
Sagittal spinal inclination (°)	1.25	3.49	−11.68	8.89
Cervical sagittal arrow (mm)	45.03	17.47	4	81
Lumbar sagittal arrow (mm)	42.21	13.18	15	76
Thoracic kyphosis (°)	42.12	8.93	23.30	65.83
Lumbar lordosis (°)	42.77	12.06	20.40	76.45
Coronal imbalance (mm)	−6.03	7.98	−23	11
Trunk imbalance (mm)	0.77	1.37	−2.10	3.07
Shoulder obliquity (mm)	3.74	10.71	−11	27
Pelvic obliquity (mm)	1.27	4.03	−9	8
Surface rotation RMS (°)	4.02	4.21	0	21.26

**Table 3:** Comparison of spinal parameters between male and female participants.

Parameter	Male (Mean ± SD)	Female (Mean ± SD)	*p*-Value
Thoracic kyphosis (°)	43.10 ± 9.16	41.28 ± 8.69	0.269
Lumbar lordosis (°)	44.21 ± 12.82	41.11 ± 11.29	0.169
Surface rotation RMS (°)	2.85 ± 3.17	5.01 ± 4.62	0.008

**Table 4:** Pearson correlation matrix between major spinal parameters.

Parameter	Kyphosis	Lordosis	Cervical Arrow	Lumbar Arrow	RMS Rotation
Kyphosis	1.00	0.53	0.77	0.51	−0.48
Lordosis	0.53	1.00	0.58	0.62	−0.32
Cervical Arrow	0.77	0.58	1.00	0.54	−0.52
Lumbar Arrow	0.51	0.62	0.54	1.00	−0.31
RMS Rotation	−0.48	−0.32	−0.52	−0.31	1.00

**Table 5:** Age-adjusted linear regression analysis predicting coronal imbalance.

Variable	β (Standardized Coefficient)	*p*-Value
Surface rotation RMS	0.38	<0.01
Age	0.12	0.09

Note: Model R^2^ = 0.21.

**Table 6:** Distribution of postural deviations based on severity thresholds.

Parameter	Normal (%)	Mild Deviation (%)	Moderate/Severe (%)
Sagittal inclination	71.4	24.5	4.1
Cervical sagittal arrow	62.2	28.6	9.2
Lumbar sagittal arrow	64.3	26.5	9.2
Thoracic kyphosis	67.3	27.6	5.1
Lumbar lordosis	65.3	28.6	6.1
Coronal imbalance	74.5	20.4	5.1
Surface rotation RMS	79.6	2.0	18.4

**Table 7:** Age-adjusted logistic regression analysis predicting rotational asymmetry (RMS > 6°).

Variable	OR	*p*-Value
Coronal imbalance	1.11	0.02
Sagittal inclination	1.07	0.03
Lumbar lordosis	1.04	0.08
Age	1.02	0.21

## Data Availability

The data presented in this study are available on request from the corresponding author. The data are not publicly available due to ethical and privacy restrictions related to human participant confidentiality.

## Abbreviations

The following abbreviations are used in this manuscript:

3D	Three-Dimensional
RMS	Root Mean Square
VP	Vertebra Prominens
DM	Dimples of Venus
SD	Standard Deviation
